# Comparison of survival in non-metastatic inflammatory and other T4 breast cancers: a SEER population-based analysis

**DOI:** 10.1186/s12885-021-07855-z

**Published:** 2021-02-06

**Authors:** Dechuang Jiao, Jingyang Zhang, Jiujun Zhu, Xuhui Guo, Yue Yang, Hui Xiao, Zhenzhen Liu

**Affiliations:** Department of breast disease, Henan Breast Cancer Center, Affiliated Cancer Hospital of Zhengzhou University & Henan Cancer Hospital, No.127, Dongming Road, Zhengzhou, 450008 China

**Keywords:** Inflammatory breast cancer (IBC); breast Cancer-specific survival (BCSS); survival rate; survival analysis;

## Abstract

**Background:**

Previous studies have reported poor survival rates in inflammatory breast cancer (IBC) patients than non-inflammatory local advanced breast cancer (non-IBC) patients. However, until now, the survival rate of IBC and other T4 non-IBC (T4-non-IBC) patients remains unexplored.

**Methods:**

Surveillance, Epidemiology, and End Results (SEER) database was searched to identify cases with confirmed non-metastatic IBC and T4-non-IBC who had received surgery, chemotherapy, and radiotherapy between 2010 and 2015. IBC was defined as per the American Joint Committee on Cancer (AJCC) 7th edition. Breast Cancer-Specific Survival (BCSS) was estimated by plotting the Kaplan-Meier curve and compared across groups by using the log-rank test. Cox model was constructed to determine the association between IBC and BCSS after adjusting for age, race, stage of disease, tumor grade and surgery type.

**Results:**

Out of a total of 1986 patients, 37.1% had IBC and mean age was 56.6 ± 12.4. After a median follow-up time of 28 months, 3-year BCSS rate for IBC and T4-non-IBC patients was 81.4 and 81.9%, respectively (log-rank *p* = 0.398). The 3-year BCSS rate in HR−/HER2+ cohort was higher for IBC patients than T4-non-IBC patients (89.5% vs. 80.8%; log-rank *p* = 0.028), and in HR−/HER2- cohort it was significantly lower for IBC patients than T4-non-IBC patients (57.4% vs. 67.5%; log-rank *p* = 0.010). However, it was identical between IBC and T4-non-IBC patients in both HR+/HER2- (85.0% vs. 85.3%; log-rank *p* = 0.567) and HR+/HER2+ (93.6% vs. 91.0%, log-rank *p* = 0.510) cohorts. After adjusting for potential confounding variables, we observed that IBC is a significant independent predictor for survival of HR−/HER2+ cohort (hazards ratio [HR] = 0.442; 95% CI: 0.216–0.902; *P* = 0.025) and HR−/HER2- cohort (HR = 1.738; 95% CI: 1.192–2.534; *P* = 0.004).

**Conclusions:**

Patients with IBC and T4-non-IBC had a similar BCSS in the era of modern systemic treatment. In IBC patients, the HR−/HER2+ subtype is associated with a better outcome, and HR−/HER2- subtype is associated with poorer outcomes as compared to the T4-non-IBC patients.

## Background

Inflammatory breast cancer (IBC) is a rare and fatal form of breast cancer. It accounts for only 2–5% of all newly diagnosed breast cancer cases and 7% of all breast cancer-specific mortality [1, 2]. Despite the lack of randomized clinical trial evidence for optimal chemotherapy in IBC, the chemotherapy regimens recommended for non-IBC are equally effective for IBC [2].

IBC is characterized by aggressive metastasis as compared to non-IBC. The Tumor Node Metastasis (TNM) classification system is widely used to distinguish IBC from non-IBC. However, until now, no specific diagnostic biomarker is available to differentiate these cancer types, except for the Tumor-Node-Metastasis (TNM) classification system. IBC was introduced under subcategory T4d by the American Joint Committee on Cancer (AJCC) in the 7th edition and refined by specifying the extent of skin involvement in IBC as one third or even more skin overlying the breast [3].

According to a previous study, survival rate beyond 5 years and the median overall survival (OS), for IBC is less than 5% and 15 months, respectively, even after the surgical or chemotherapeutic treatment [4]. The multidisciplinary therapeutic regimen of breast cancer primarily includes neoadjuvant chemotherapy, surgery, radiotherapy, and hormone therapy. It substantially improves the survival rate of patients with IBC and non-IBC. A recent study reported, 71 and 31%, 5 and 10 year OS rates, respectively, in IBC patients [5] [6].

As per the previous studies, IBC subjects have a poor survival rate than the subjects with locally advanced non-IBC [1, 7–9]. Based on the 2004–2007 SEER registry data, Dawood et al. [10] had reported that 2-year BCSS of non-IBC patients is higher than the IBC patients (91% vs. 84%; *P* < 0.0001) who received anthracycline- and taxane-based drugs. Locally advanced breast cancer in stage III B/C were included in this study; however, some non-T4 breast cancers were also included in stage III C breast cancer and this study had no information about the molecular subtypes of breast cancer.

Biological behavior and molecular characteristics of IBC differs from non-IBC [11, 12], and IBC and other T4-non-IBC have similar breast tumor burden. In this study, we determined the difference in survival rate between IBC and T4-non-IBC based on a large cohort of samples. It will extend our understanding of the characteristics associated with IBC. Besides, it may help the clinicians to formulate a more effective therapeutic regimen for IBC.

## Methods

### Patient population

In this study, the National Cancer Institute’s SEER program data were analyzed. The dataset included demographics, cancer incidences, tumor characteristics, treatment, and survival outcomes of around 26% of the US population [13]. We analyzed the18-SEER database, which was released in April 2018. The inclusion criteria was pathologically confirmed non-metastatic T4 breast cancer cases with single primary malignancy and cancer subtype information. Cases diagnosed between 2010 and 2015, which met the inclusion criteria, and had undergone trimodality treatment (chemotherapy, surgery, and radiation therapy) were included in this study.

SEER initiated HER2 data collection in 2010, and thus, exclusion criteria included diagnosis before 2010. The AJCC 7th edition criteria were used to characterize the T4 as well as IBC, and the IBC cases were characterized as T4d from the SEER database.

### Statistical analysis

Patients were categorized as IBC and T4-non-IBC, and patient’s characteristics were compared using the χ2 test. The follow-up cutoff was set to December 31, 2015. BCSS was estimated from the date of initial diagnosis until the date of breast cancer-related death. Kaplan-Meier curves were used to estimate BCSS. Two groups were compared by using the log-rank test. Cox proportional hazards models were constructed to determine the association between IBC and subtype-specific survival outcomes after adjusting the patient’s characteristics. The final multivariable model included variables that were based on clinical significance rather than statistical significance and included age, race, stage of disease, tumor grade, surgery type and breast cancer type. Results were expressed in hazard ratios (HRs) and 95% CIs.

All tests were two-sided. Statistical analyses were carried out by using SPSS version 22.0 (IBM Corporation, NY, USA). *P*-value *<* 0.05 was considered statistically significant.

## Results

### Patient baseline characteristics

A total of 1986 patients with non-metastatic T4 breast cancer who were enrolled in the SEER database between January 2010 till December 2015 were included in this study. Out of 1986 patients, 737 and 1249 had IBC and non-IBC, respectively; besides, 190 and 1796 patients underwent a partial and total mastectomy, respectively. The clinical and tumor characteristics of IBC and non-IBC patients were compared as demonstrated in Table 1**.** The outcome indicated that most of the IBC patients were younger during the onset of presentation, white people, high histological grade, HER2-positive, ER- and PR-negative than non-IBC patients; however, the cases in both groups showed similar clinical-staging. Besides, partial mastectomy rate in IBC patients was less than T4-non-IBC patients (4.2% vs. 12.7%; *P* < 0.001).

**Table 1:** Patient Characteristics Stratified by Breast Cancer Type

	Overall (*n* = 1986)	T4-non-IBC (*n* = 1249)	IBC (*n* = 737)	*P*
Age,y,No. (%)				0.015
< 50	614(30.9)	362 (29.0)	252 (34.2)	
≥ 50	1372 (69.1)	887 (71.0)	485 (65.8)	
Race,No. (%)				< 0.001
Black	358 (18.0)	255 (20.4)	103 (14.0)	
White	1432 (72.1)	859 (68.8)	573 (77.7)	
Other	196 (9.9)	135 (10.8)	61 (8.3)	
Tumor stage, No. (%)				0.197
IIIB	1622 (81.7)	1032 (82.6)	590 (80.1)	
IIIC	364 (18.3)	217 (17.4)	147 (19.9)	
Grade,No. (%)				< 0.001
1/2	659 (33.2)	459 (36.7)	200 (27.1)	
3	1220 (61.4)	734 (58.8)	486 (65.9)	
Unknown	107 (5.4)	56 (4.5)	51 (6.9)	
Surgery,No. (%)				< 0.001
Partial mastectomy	190 (9.6)	159 (12.7)	31 (4.2)	
Mastectomy	1796 (90.4)	1090 (87.3)	706 (95.8)	
ER status, No. (%)				< 0.001
Positive	1214 (61.1)	814 (65.2)	400 (54.3)	
Negative	772 (38.9)	435 (34.8)	337 (45.7)	
PR status, No. (%)				< 0.001
Positive	984 (49.5)	665 (53.2)	319 (43.3)	
Negative	1002 (50.5)	584 (46.8)	418 (56.7)	
Her2 status, No. (%)				< 0.001
Positive	672 (33.8)	372 (29.8)	300 (40.7)	
Negative	1314 (66.2)	877 (70.2)	437 (59.3)	
Tumor subtype, No. (%)				< 0.001
HR+/HER2–	871 (43.9)	612 (49.0)	259 (35.1)	
HR+/HER2+	397 (20.0)	233 (18.7)	164 (22.3)	
HR−/HER2+	275 (13.8)	139 (11.1)	136 (18.5)	
HR−/HER2–	443 (22.3)	265 (21.2)	178 (24.2)	

### BCSS of IBC and T4-non-IBC patients

**Fig. 1:**
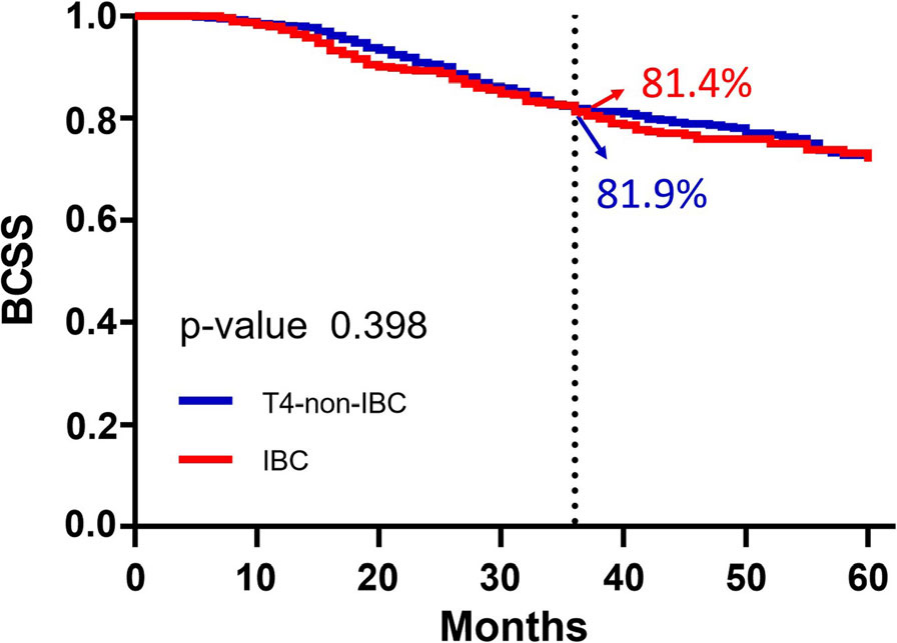
BCSS of IBC and T4-non-IBC patients

During a median follow-up of 28 months (range 0–60), a total of 293 breast cancer-related deaths were observed, out of which T4-non-IBC and IBC accounted for 176 and 117 deaths, respectively. Three-years BCSS was similar for IBC and T4-non-IBC patients (81.4% vs. 81.9%; log-rank *p* = 0.398) **(**Fig. 1**)**. Out of all the T4 patients included in this study, improved survival was observed in cases which were HR+*/*HER2+, non-black or white with an early-stage tumor stage, and lower tumor grade **(**Table 2**).** HR+*/*HER2+ subtype was significantly associated with a better BCSS (HR: 0.557, 95% CI: 0.349–0.890; *P* = 0.014) and HR−*/*HER2- subtype with a poorer BCSS (HR: 2.819, 95% CI: 2.082–3.818; *P <* 0.001), while BCSS were identical for HR−*/*HER2+ subtype (HR: 1.272, 95% CI: 0.880–1.837; *P* = 0.200) as per the multivariate analysis using HR+*/*HER2- subtype as reference. Table 2 depicts the additional factors associated with 3-years BCSS of IBC and T4-non-IBC.

**Table 2:** Three-Years Breast Cancer-Specific Survival and Multivariable Model (Adjusted for SEER Registry)

	Univariate analysis		multivariate analysis		
	3 years-BCSS, (%)	*P*	HR	95%CI	*P*
Type of cancer		0.398			
IBC	81.4		1		
T4-non-IBC	81.9		1.125	0.885–1.430	0.337
Age,y		0.532			
< 50	82.8		1		
≥ 50	81.2		1.049	0.816–1.347	0.711
Race		0.013			
Black	77.3		1		
White	81.9		0.906	0.684–1.202	0.495
Other	89.0		0.583	0.353–0.965	0.036
Tumor stage		< 0.001			
IIIB	84.2		1		
IIIC	70.8		2.378	1.847–3.061	< 0.001
Grade		< 0.001			
1/2	90.4		1		
3	77.3		1.84	1.355–2.500	< 0.001
Unknown	79.6		1.919	1.077–3.417	0.027
Surgery		0.087			
Partial mastectomy	88.7		1		
Mastectomy	81.1		1.305	0.806–2.112	0.279
Tumor subtype		< 0.001			
HR+/HER2–	85.2		1		
HR+/HER2+	92.1		0.485	0.318–0.740	0.001
HR−/HER2+	85.2		0.867	0.588–1.279	0.472
HR−/HER2–	63.6		2.164	1.652–2.836	< 0.001

### Analysis of BCSS by breast cancer moleculor subtypes

**Fig. 2:**
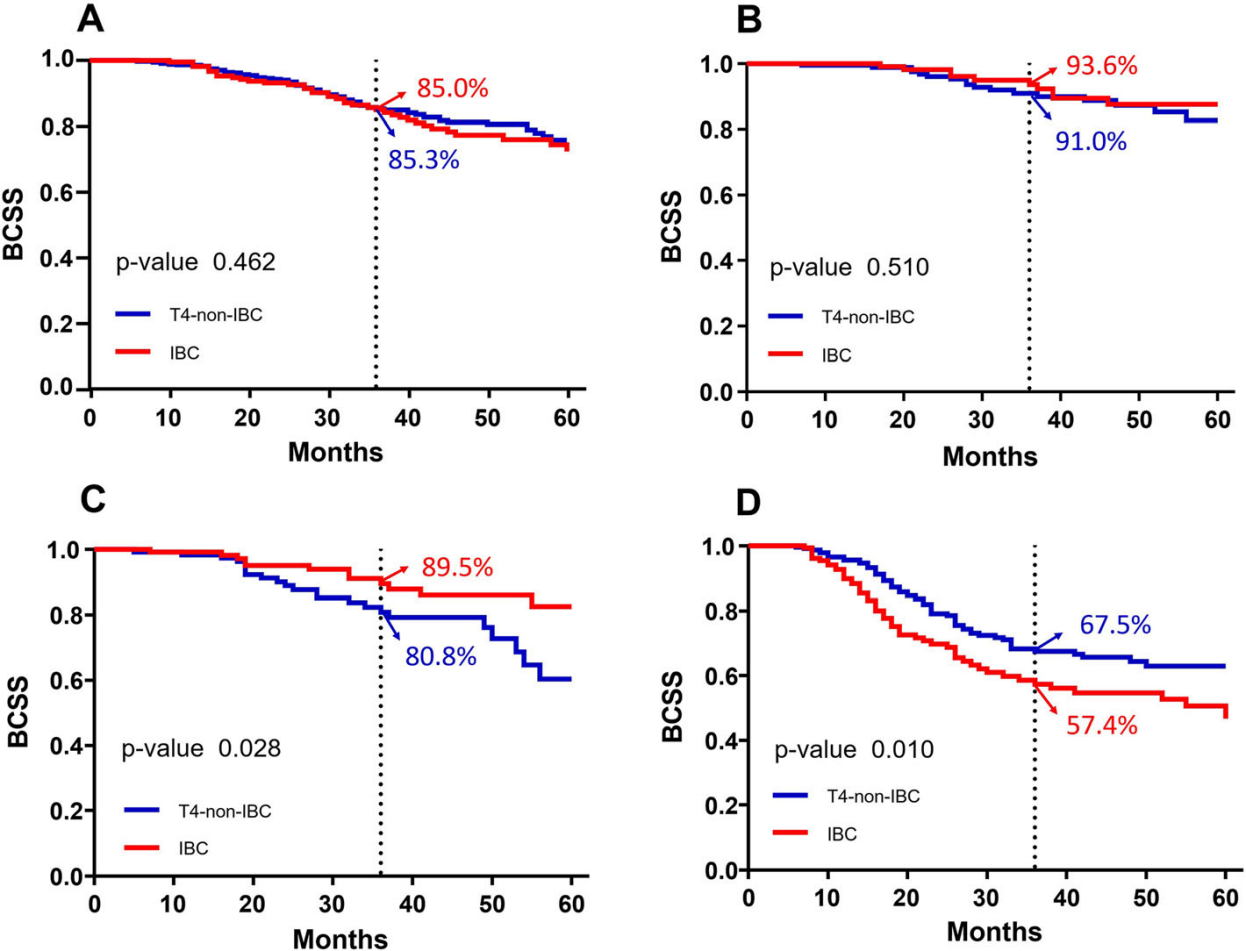
Breast cancer-specific survival for patients with IBC and T4-non-IBC stratified by breast cancer moleculor subtype. **a** HR+/HER2-;(**b**)HR+/HER2+;(**c**)HR−/HER2+;(**d**)HR−/HER2-. HER2 indicates human epidermal growth factor receptor 2; HR, hormone receptor; IBC,Inflammatory breast cancer; T4-non-IBC, T4 noninflammatory breast cancer

The clinicopathological features of breast cancer with different molecular subtypes are depicted in Table 3**.** The 3-years BCSS was higher for patients with HR−/HER2+ IBC than HR−/HER2 + T4-non-IBC patients (89.5% vs. 80.8%; *P* = 0.028), but lower for HR−/HER2- IBC than HR−/HER2- T4-non-IBC patients (57.4% vs. 67.5%; *P* = 0.010). In the rest of the tumor subtypes, BCSS did not differ significantly between IBC and T4-non-IBC patients **(**Fig. 2**)**.

**Table 3:** Patient Characteristics Stratified by Breast Cancer Moleculor Subtype

	Overall (*n* = 1986)	HR+/HER2- (*n* = 871)	HR+/HER2+ (*n* = 150)	HR−/HER2+ (*n* = 275)	HR−/HER2- (*n* = 443)	*P*
Age,y,No. (%)						0.736
< 50	614(30.9)	264 (30.3)	132 (33.2)	84 (30.5)	134 (30.2)	
≥ 50	1372 (69.1)	607 (69.7)	265 (66.8)	191 (69.5)	309 (69.8)	
Race,No. (%)						< 0.001
Black	358 (18.0)	133 (15.3)	62 (15.6)	44 (16.0)	119 (26.9)	
White	1432 (72.1)	639 (73.4)	298 (75.1)	208 (75.6)	287 (64.8)	
Other	196 (9.9)	99 (11.4)	37 (9.3)	23 (8.4)	37 (8.4)	
Tumor stage, No. (%)						0.218
IIIB	1622 (81.7)	728 (83.6)	316 (79.6)	225 (81.8)	353 (79.7)	
IIIC	364 (18.3)	143 (16.4)	81 (20.4)	50 (18.2)	90 (20.3)	
Grade,No. (%)						< 0.001
1/2	659 (33.2)	299 (45.8)	150 (37.8)	56 (20.4)	54 (12.2)	
3	1220 (61.4)	428 (49.1)	223 (56.2)	201 (73.1)	368 (83.1)	
Unknown	107 (5.4)	44 (5.1)	24 (6.0)	18 (6.5)	21 (4.7)	
Surgery,No. (%)						0.104
Partial mastectomy	190 (9.6)	93 (10.7)	33 (8.3)	17 (6.2)	47 (10.6)	
Mastectomy	1796 (90.4)	778 (89.3)	364 (91.7)	258 (93.8)	396 (89.4)	
Breast cancer type, No. (%)						< 0.001
IBC	737 (37.1)	259 (29.7)	164 (41.3)	136 (49.5)	178 (40.2)	
T4-non-IBC	1249 (62.9)	612 (70.3)	233 (58.7)	139 (50.5)	265 (59.8)

Furthermore, we constructed four Cox multivariate regression models to investigate the specific interaction effects of the cancer type (IBC or T4-non-IBC) with breast cancer subtypes **(**Table 4**).** The models were adjusted for age, grade, hormone receptor status, tumor stage, HER-2 status, and surgery. After adjusting for these variables, we observed that the hazard of breast cancer-specific deaths was significantly reduced in HR−/HER2+ IBC patients (HR: 0.442; 95% CI: 0.216–0.902; *P* = 0.025) and significantly increased in HR−/HER2- IBC patients (HR: 1.738; 95% CI: 1.192–2.534; *P* = 0.004) as compared to T4-non-IBC patients.

**Table 4:** Cox Proportional Hazards Regression Model Analysis of Breast Cancer-Specific Survival by Breast Cancer Moleculor Subtype

	HR+/HER2-			HR+/HER2+			HR−/HER2+			HR−/HER2-		
	HR	95%CI	*P*	HR	95%CI	*P*	HR	95%CI	*P*	HR	95%CI	*P*
Age												
< 50	1.000			1.000			1.000			1.000		
≥ 50	0.870	0.583–1.299	0.497	0.854	0.388–1.880	0.695	1.573	0.707–3.500	0.267	1.221	0.813–1.836	0.336
Race												
Black	1.000			1.000			1.000			1.000		
White	0.738	0.466–1.169	0.196	1.395	0.473–4.115	0.547	1.052	0.398–2.779	0.918	0.870	0.570–1.327	0.517
Other	0.387	0.174–0.860	0.020	0.446	0.049–4.050	0.473	0.783	0.182–3.364	0.742	0.795	0.362–1.742	0.566
Tumor stage												
IIIB	1.000			1.000			1.000			1.000		
IIIC	2.444	1.607–3.716	< 0.001	1.800	0.776–4.173	0.171	2.184	0.950–5.024	0.066	2.756	1.863–4.075	< 0.001
Grade												
1/2	1.000			1.000			1.000			1.000		
3	2.241	1.473–3.407	< 0.001	2.306	0.914–5.816	0.077	0.611	0.281–1.331	0.215	2.221	1.115–4.422	0.023
Unknown	2.543	1.055–6.128	0.038	2.534	0.494–13.01	0.265	0.689	0.146–3.247	0.637	2.007	0.662–6.085	0.218
Surgery												
Partial mastectomy	1.000			1.000			1.000			1.000		
Mastectomy	0.828	0.436–1.574	0.565	1.444	0.189–11.04	0.724	1.115	0.254–4.890	0.885	2.453	0.898–6.700	0.080
Breast cancer type												
T4-non-IBC	1.000			1.000			1.000			1.000		
IBC	1.029	0.690–1.534	0.888	0.678	0.307–1.499	0.337	0.442	0.216–0.902	**0.025**	1.738	1.192–2.534	**0.004**

## Discussion

The objective of the current study was to analyze the difference in survival rate between the IBC and T4-non-IBC patients and correlate this outcome with the molecular subtypes of breast cancer. The analysis revealed that there was no difference in 3-year BCSS between IBC and T4-non-IBC patients who had undergone trimodality treatment. Besides, in the HR−/HER2+ group, the 3-year BCSS rate was higher in IBC patients than T4-non-IBC patients and vice-versa in the HR−/HER2- group. There was no significant difference in the 3-year BCSS rate between IBC and T4-non-IBC in HR+/HER2- and HR−/HER2+ groups.

Dawood et al. [10] analyzed the IBC and non-IBC of IIIB/C cases from the SEER database registered during 2004–2007 and found that the risk of death increased by 43% in IBC patients as compared to non-IBC patients (HR = 1.43; CI: 1.10–1.86; *P* = 0.008). We limited the study to people with T4 breast cancer, and the conclusion is entirely different. The outcomes of our analysis indicated that the 3-year BCSS of IBC and T4-non-IBC cases were 81.4 and 81.9%, respectively (log-rank *p* = 0.398). The plausible reason for the difference in the previous study and the current study may be due to the following reasons. Firstly, Dawood’s study included IIIC breast cancer cases with different T stages, and so, breast tumor load might have impacted the prognosis of patients. In our study, when we limited the control population to T4 breast cancer cases, whose breast tumor load was similar to IBC, we did not observe any difference in the survival rate between the two groups. It is in line with the study by Anya et al., a clinical study with a small cohort of samples [5].

Secondly, in the studies by Dawood et al. and others, chemotherapy was not an inclusion criterion. Although we could not accurately estimate the number of cases from the SEER database who did not receive chemotherapy; however, the effect of chemotherapy on locally advanced breast cancer, especially IBC, was apparent. In the current study, we did not include the participants whose chemotherapy registration information specified: “No/Unknown.” It minimized the difference in survival caused by different chemotherapy acceptance rates.

Thirdly, as per our speculation, the most significant explanation for the differences in the outcome of current and previous studies might be due to the drug trastuzumab. NOAH studies have confirmed that trastuzumab, in addition to chemotherapy, treats locally advanced breast cancer effectively, including IBC [14]. However, before 2006, trastuzumab was not recommended for the treatment of early breast cancer. Cases included in Dawood’s study (2004–2005) did not receive trastuzumab in the adjuvant or neoadjuvant phase. Besides, the follow-up deadline for this study was December 31, 2007, and the cases included between 2006 and 2007 were statistically insignificant to estimate the 2-year survival rate due to the short follow-up time. And all the cases we included were diagnosed in and after 2010. Thus, we speculate that targeted therapy with trastuzumab could be a substantial underlying reason for the differences in the outcome of our and previous studies.

The application of targeted therapy can affect the differences in the conclusions of the current study due to a couple of reasons. Firstly, the proportion of HER2-positive breast cancer in the IBC group was higher than the locally advanced breast cancer in non-IBC. In this study, the proportion of HER2-positive breast cancer in the IBC group (40.7%) was higher than the T4-non-IBC breast cancer group (29.8%) (*P* < 0.01), which is consistent with the previous studies [15, 16]. Thus, the IBC group was more likely to receive targeted therapy. Secondly, trastuzumab targeted therapy benefitted IBC patients more than the patients with locally advanced breast cancer non-IBC. In the NOAH study, the use of trastuzumab during adjuvant and neoadjuvant therapy increased the 5-year OS of patients with IBC from 44 to 74% (HR = 0.38, 95% CI: 0.15–0.95), and locally advanced breast cancer in non-IBC from 68 to 73% (HR = 0.80, 95% CI: 0.48–1.32) [14].

For the first time in this study, we reported that in HR−/HER2+ subtype breast cancer, the 3-year BCSS rate of IBC patients was higher than that of T4-non-IBC patients (89.5%% vs. 80.8%; log-rank *p* = 0.028) by analyzing different subgroups. After multivariate correction of the SEER dataset, the risk of breast cancer specific death was found to be 55.8% lower in IBC group (HR: 0.442, 95% CI: 0.216–0.902). Therefore, we speculate that the meaning of HER2 overexpression in IBC and non-IBC was not exactly the same.

Iwamoto et al. [17] compared gene expression patterns between IBC and non-IBC locally advanced breast cancer with different molecular subtypes and found that HER2 positive IBC and non-IBC breast cancer have different gene expression patterns. However, the correlation between the difference in gene expression patterns and sensitivity of HER2 targeted therapy needs further in-depth investigation.

The 3-year BCSS rate of HR−/HER2- subtype IBC patients is lower than the non-IBC patients with the poorest prognosis when compared to other subtypes of IBC. Nakhlis et al. [18] analyzed 181 patients with IBC who received anthracyclines and taxanes as a part of neoadjuvant therapy and found that the TN phenotype was associated with the shortest recurrence time. It indicates that HR−/HER2- IBC might be a unique type of breast cancer.

The present study, however, is subject to certain limitations. Firstly, it is a retrospective study, and so it has inherent limitations of the retrospective study. Secondly, detailed treatment information in the SEER database was not available. Besides, specific chemotherapy plans, time of chemotherapy implementation, radiotherapy plan and radiation dose, targeted treatment, endocrine therapy implementation, and program, were not included in the SEER dataset, which might have impacted the outcome of the current study. Since the IBC accounts for only 2–5% of total breast cancer cases, the biggest advantage of this study is its big sample size, which makes even the small differences observed in this study statistically significant, specifically the analysis of subgroups.

## Conclusion

In conclusion, the current study showed that in the era of targeted therapy for HER2, there is no difference in BCSS between IBC and T4-non-IBC. Although the prognosis of IBC has improved,the overall prognosis of IBC remains poor, specifically a subtype of HR−/HER2-. Thus, it may be necessary to explore new treatment options to prolong the survival of patients with HR−/HER2- IBC. Besides, more intensive follow-up to detect recurrence of cancer in the early stage is also needed. It necessitates the further study of the HER2 positive IBC at the molecular level to unravel the underlying reason for its sensitivity to targeted therapy and the difference in molecular expression between IBC and the same subtype of T4-non-IBC.

## Data Availability

The datasets used and/or analyzed during the current study are available from the corresponding author on reasonable request.

## Abbreviations

IBC: Inflammatory breast cancer
BCSS: Breast cancer-specific survival
non-IBC: Non-inflammatory local advanced breast cancer
AJCC: American joint committee on cancer
TNM: Tumor node metastasis
OS: Overall survival
HR: Hazard ratio
